# Guillain-Barré Syndrome Presenting With Bulbar Symptoms: A Case Report

**DOI:** 10.7759/cureus.94577

**Published:** 2025-10-14

**Authors:** Agbonmwanre E Osayagbon, Abdulmumini Shehu, Kshitija Ravindra Bhutkar, Muhammad A Butt, Aliyu O Olaniyi

**Affiliations:** 1 Geriatrics, Stepping Hill Hospital, Manchester, GBR; 2 Stroke Medicine, Stepping Hill Hospital, Manchester, GBR; 3 General Medicine, Stockport NHS Foundation Trust, Manchester, GBR; 4 Medicine, Stepping Hill Hospital, Manchester, GBR

**Keywords:** acute flaccid paralysis, bulbar symptoms, cranial nerve, gbs, guillain-barré syndrome, neuromuscular disorder

## Abstract

Guillain-Barré syndrome (GBS) is an acute, immune-mediated neuropathy characterized by rapidly progressive, symmetrical weakness, areflexia, and variable sensory loss. It is often triggered by preceding respiratory or gastrointestinal infection through a mechanism of molecular mimicry, leading to demyelination or axonal injury. Diagnosis is supported by cerebrospinal fluid albumin-cytologic dissociation and nerve conduction studies. Early initiation of intravenous immunoglobulin or plasma exchange, alongside vigilant supportive care, reduces morbidity and prevents complications such as respiratory failure and autonomic instability. In this case report, we describe a patient presenting with vague and non-specific symptoms, including dizziness and lower back pain while walking, generalized weakness, and early bulbar symptoms such as difficulty swallowing and slurred speech. These early manifestations, though subtle, gradually progressed and prompted further evaluation, highlighting the stepwise diagnostic process, targeted investigations, and the multidisciplinary team approach that guided his medical management of GBS.

## Introduction

Guillain-Barré syndrome (GBS) is an acute, immune-mediated destruction of multiple peripheral nerves and their spinal roots [1]. This condition is marked by a sudden onset of muscle weakness and the absence of reflexes, giving rise to its hallmark features of ascending flaccid paralysis, loss of reflexes, and varying degrees of sensory and autonomic dysfunction [2]. It is regarded as a neurological emergency because the disease can rapidly progress to autonomic instability and respiratory failure [3]. The underlying mechanism involves an abnormal autoimmune response that targets the peripheral nerves, often occurring after a preceding infection such as *Campylobacter jejuni* [4]. Other implicated organisms include *Mycoplasma pneumoniae*, cytomegalovirus, Epstein-Barr virus, and influenza virus [5,6]. There have been rare cases associated with vaccinations, but the risk is very low and outweighed by vaccine benefits [7]. 

## Case presentation

A 78-year-old man presented to the emergency department with dizziness, difficulty swallowing, and lower back pain while walking. His partner, who accompanied him, reported that she had found him on the floor before hospital admission. The patient described a one-week history of progressively worsening back pain radiating along the spine, associated with declining mobility. He agreed to attend the hospital for further evaluation to exclude fractures and possible spinal cord pathology.

His past medical history included bowel cancer (2021), managed with high anterior resection, anastomosis, and adjuvant chemotherapy, with no evidence of recurrence; Raynaud’s phenomenon; Dupuytren’s contracture; and pleomorphic adenoma of the parotid gland. He lived independently, was an ex-smoker (cessation 30 years before), consumed alcohol socially, and was fully independent in activities of daily living. He had recently returned from a cruise three weeks earlier, reporting no post-travel symptoms.

On initial assessment, he was alert, oriented, and in no acute distress. Blood pressure was elevated at 196/110 mmHg. Cardiovascular, respiratory, and abdominal examinations, including digital rectal examination, were unremarkable. Neurological evaluation demonstrated normal upper limb power (5/5), reduced lower limb power (4/5), and diminished lower limb reflexes, without urinary or fecal incontinence. An initial working impression of dizziness, likely secondary to benign paroxysmal positional vertigo, and lower back pain, probably due to osteoarthritis, was made. The patient was admitted and commenced on low-molecular-weight heparin (as per hospital protocol), analgesia, betahistine, and antihypertensives for elevated blood pressure. He remained in the acute medical unit for 24 hours before transfer to the medical ward.

Within 24 hours of transfer, his clinical condition deteriorated. He developed new-onset drowsiness, confusion, oxygen desaturation (<94% on room air), slurred speech, quadriparesis, and urinary retention necessitating catheterization. Supplemental oxygen was commenced at 4 L/min via nasal cannula. Neurological re-examination revealed marked weakness with lower limb power of 0/5 and upper limb power of 1/5, accompanied by reduced reflexes but preserved sensation. Stroke was initially considered, given the elevated blood pressure, slurred speech, and urinary incontinence. Cervical myelopathy was also considered due to earlier findings of lower limb weakness. His National Institutes of Health Stroke Scale score was calculated at 25. An urgent referral was made to the stroke team, who recommended MRI of the brain and spine, CT angiography of the aorta, and initiation of high-dose aspirin.

Neuroimaging, including MRI brain, MRI spine, and CT angiography, was performed and subsequently reviewed at a neuroradiology meeting. These investigations were reported as normal, effectively excluding stroke and spinal cord compression. Given the clinical deterioration, prompt referrals were made to both the neurology team and the intensive care unit (ICU), as the patient was for full resuscitation. In ICU, he maintained adequate oxygen saturations on nasal cannula oxygen supplementation. The neurology team recommended lumbar puncture alongside empirical antibiotic and antiviral therapy for suspected encephalitis.

Routine blood investigations, including viral screening, renal function, and hematological parameters, were essentially normal. Cerebrospinal fluid (CSF) analysis revealed albumin-cytologic dissociation, confirming the diagnosis of GBS, with a GBS disability score of 4. The patient was commenced on intravenous immunoglobulin (IVIG) therapy. Laboratory results are summarized in Table 1 (CSF analysis), Table 2 (full blood count, renal function, viral serology, anti-neutrophil cytoplasmic autoantibodies, and immunoglobulin profile--all within normal reference ranges), and Table 3 (GBS disability scale). Blood cultures showed no microbial growth after incubation, further excluding alternative infectious diagnoses.

**Table 1:** CSF analysis results CSF: Cerebrospinal fluid; IgG: Immunoglobulin G

Parameter	Value	Normal range
CSF total protein	1.70 g/L	0.15-0.60 g/L
CSF glucose	3.2 mmol/L	2.2-4.4 mmol/L
CSF lactate	1.9 mmol/L	1.4-2.6 mmol/L
Plasma glucose	8.2 mmol/L	3.3-6.0 mmol/L
CSF IgG	0.181 g/L	0.58-0.74 g/L
CSF albumin	1.021 g/L	0.10-0.45 g/L
IgG	6.6 g/L	6.0-16.0 g/L
Serum albumin	36 g/L	34-47 g/L

**Table 2:** Blood investigation results GFR: Glomerular filtration rate; MPO: Myeloperoxidase; PCV: Packed cell volume; PR3: Proteinase 3.

Laboratory parameter	Result	Range
White cell count	6.5 x 10^9^/L	3.7-11.0 x 10^9^/L
Hemoglobin	114 g/L	130-180 g/L
Mean cell volume	96.7 fl	76.0-100.0 fl
Platelet count	285 x 10^9^/L	150-450 x 10^9^/L
Neutrophil	3.4 x 10^9^/L	1.7-7.5 x 10^9^/L
Lymphocyte	2.2 x 10^9^/L	1.0-4.5 x 10^9^/L
C-reactive protein	6.8 mg/L	0.0-10.0 mg/L
Hematocrit (PCV)	0.33 L/L	0.40-0.54 L/L
Serum magnesium	0.74 mmol/L	0.70-1.00 mmol/L
Serum calcium (adjusted)	2.49 mmol/L	2.20-2.60 mmol/L
Serum sodium	133 mmol/L	133-146 mmol/L
Serum potassium	4.2 mmol/L	3.5-5.3 mmol/L
Serum urea	10.2 mmol/L	2.5-7.8 mmol/L
Serum creatinine	68 umol/L	62-65 umol/L
Estimated GFR	>90 mL/min	>90 mL/min
Serum phosphate	1.03 mmol/L	0.80-1.50 mmol/L
MPO	<0.2 AI	0.0-0.9 AI
PR3	<0.2 AI	0.0-0.9 AI
Immunoglobulin G	7.1 g/L	6.0-16.0 g/L
Immunoglobulin A	1.7 g/L	0.8-4.0 g/L
Immunoglobulin M	0.7 g/L	0.5-2.0 g/L
Hepatitis C antibody	Negative	-
Hepatitis B surface antigen	Negative	-
HIV 1+2 and p24 antigen	Not detected	-

**Table 3:** GBS disability scale [1] GBS: Guillain-Barré syndrome.

Grade	Description
0	Healthy, no symptoms or functional signs of neuropathy
1	Minor symptoms and signs of neuropathy but able to run
2	Able to walk 10 meters or more without assistance but unable to run
3	Able to walk 10 meters across an open space with assistance (support, cane, or walker)
4	Bedridden or chair-bound (unable to walk, even with help)
5	Requires assisted ventilation (intubation or tracheostomy)
6	Dead

**Figure 1:**
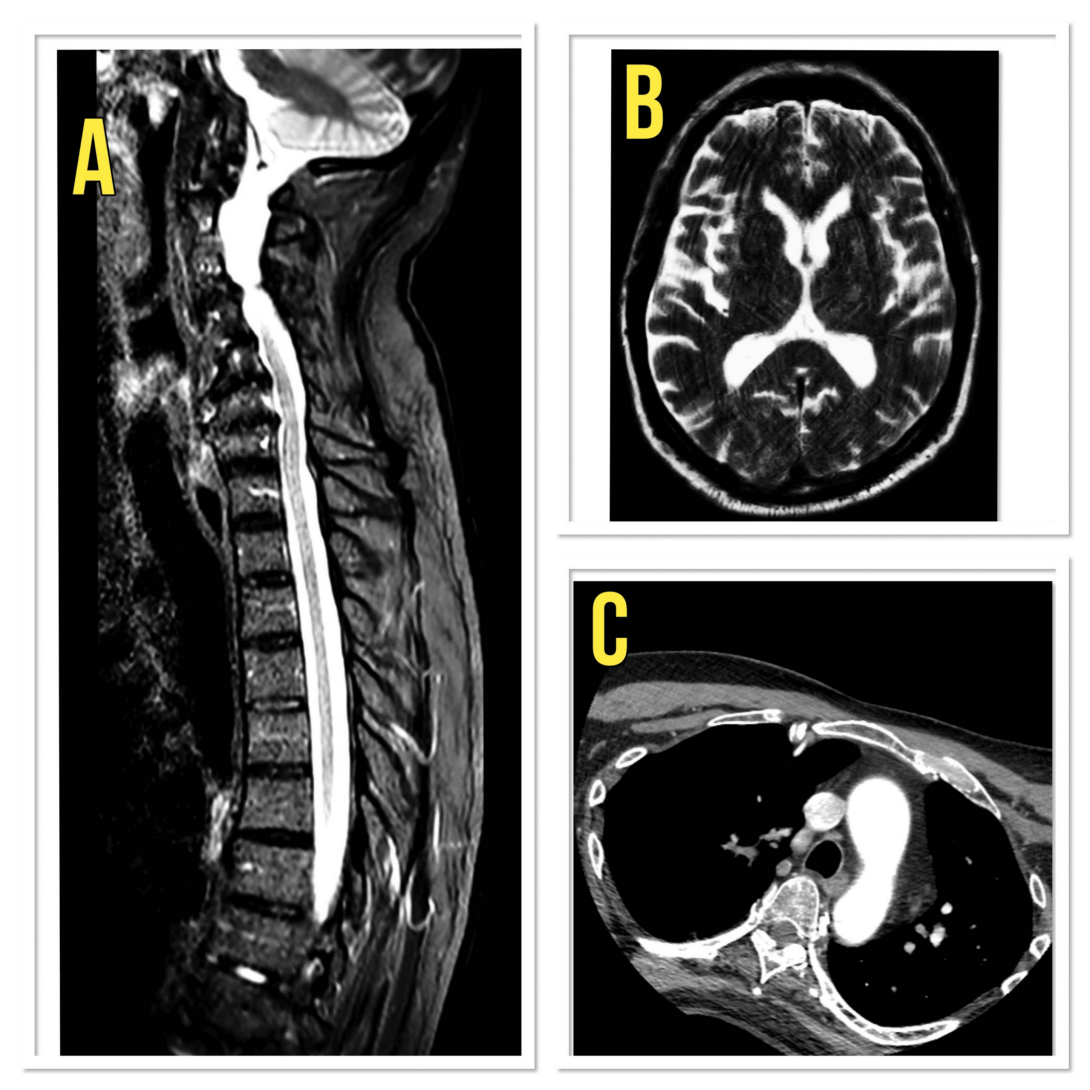
MRI whole spine, MRI head, and CT angiogram aorta of the patient (A) MRI whole spine, (B) MRI head, and (C) CT angiogram aorta. All investigations were reported as normal, and no pathology was identified in the imaging related to the symptoms of the patient.

Imaging studies, including CT angiography, MRI of the head, and MRI of the whole spine, are shown in Figure 1. Although the MRI brain was affected by motion artefact, it was interpreted as grossly normal by the radiology department. Collectively, these findings supported the diagnosis of GBS while excluding alternative neurological or structural causes.

While in the ICU, nutrition was maintained via nasogastric feeding. He was not intubated, as he maintained oxygen saturation with a nasal cannula. He was given IVIG for five days as per treatment protocol and was stepped down to the medical ward from the ICU, as he showed remarkable improvement. Over the following weeks, in the ward, he demonstrated significant improvement in his clinical and physical condition. Speech and language therapy assessment enabled progression to a modified diet, while physiotherapy facilitated mobilization with a walking aid. After eight weeks of inpatient treatment and multidisciplinary rehabilitation, he was discharged to a neurorehabilitation unit for ongoing neurorehabilitation for some residual speech abnormality and lower limb strengthening exercises.

## Discussion

In this case study, the patient was an elderly male who presented with lower back pain and reduced mobility. GBS is a relatively rare but clinically significant neurological disorder, with an estimated annual incidence of 1-2 cases per 100,000 population, reported predominantly in Europe and North America [8]. Epidemiological studies have noted seasonal variation in certain regions, often associated with outbreaks of gastrointestinal or respiratory infections. The incidence is lowest among children (1 per 100,000), shows a male predominance with a ratio of approximately 1.5:1, and increases with advancing age [9]. Although GBS may occur at any age, it is most frequently observed in adults between 30 and 50 years. The present case, involving an elderly male, aligns with these established demographic trends.

A notable surge in GBS cases was documented during the Zika virus outbreaks in Latin America, supporting the hypothesis that viral infections can play a pivotal role in triggering the autoimmune cascade [10]. While GBS is typically preceded by an infectious illness and characterized by ascending weakness, its absence does not preclude diagnosis, as exemplified in this case. The patient initially presented with non-specific symptoms, including dizziness, back pain, and gait instability, which complicated early recognition. However, rapid progression to quadriparesis, bulbar dysfunction, and urinary retention necessitated urgent neurological evaluation.

Advances in research have considerably enhanced understanding of the immunopathogenesis, diagnostic evaluation, and therapeutic approaches to GBS. The most widely accepted mechanism of immunopathogenesis is molecular mimicry, wherein antibodies directed against infectious agents cross-react with gangliosides on peripheral nerves, leading to demyelination or axonal degeneration [11]. Clinically, GBS manifests in distinct subtypes, most notably acute inflammatory demyelinating polyradiculoneuropathy, which is predominant in Europe and North America. The axonal variants, acute motor axonal neuropathy and acute motor and sensory axonal neuropathy, are more common in Asia and Latin America [11]. Electrophysiological studies are critical for differentiating these subtypes, although they were not performed in the present case, as it takes time to carry out these investigations [12,13]. Diagnosis of GBS is primarily based on clinical features and CSF analysis demonstrating albumino-cytologic dissociation, supported by electrophysiological studies when available.

According to the Brighton criteria, a diagnosis is established through the integration of characteristic clinical findings and confirmatory investigations [14]. In this patient, the key diagnostic finding was albuminocytologic dissociation on CSF analysis, characterized by elevated protein with a normal white blood cell count, which strongly supported the diagnosis and prompted the timely initiation of IVIG therapy. The case further illustrates how GBS can mimic other acute neurological emergencies such as stroke or spinal cord compression. Imaging of the brain and spine excluded cord compression, intracranial hemorrhage, and fractures, as reported by radiology, underscoring the importance of broad differential diagnosis and early specialist input. Recent research has highlighted potential biomarkers for early diagnosis and prognosis, including anti-ganglioside antibodies, which are associated with specific subtypes and adverse outcomes [15]. 

With respect to management, IVIG and plasmapheresis are established as first-line therapies, both demonstrating equivalent efficacy in expediting recovery and reducing disease severity [16]. In this case, IVIG was administered and proved effective in the patient’s recovery. In contrast, corticosteroids, once considered a therapeutic option, have not demonstrated consistent benefit in randomized controlled trials [17]. Optimal management of GBS requires a multidisciplinary approach, involving neurologists, intensive care specialists, speech and language therapists, dietitians, and physiotherapists. Early initiation of immunotherapy, coupled with proactive supportive measures such as airway protection, nutritional support, and prevention of secondary complications, is essential for improving outcomes. The long-term prognosis in GBS is generally favorable, with most patients achieving substantial recovery within several months. Nevertheless, up to 20% of patients may experience persistent weakness, and 5%-10% may die from complications such as respiratory failure or cardiac arrhythmias [18].

## Conclusions

This case demonstrates how GBS can present with vague, non-specific symptoms and progress rapidly to severe neurological compromise. While most cases of GBS follow a recognized cause, the syndrome can also occur in the absence of identifiable triggers. A high index of suspicion, prompt investigation, and early multidisciplinary management are essential to improving patient outcomes. Clinicians should remain vigilant for the possibility of GBS, even in the absence of a classic preceding infection, as early recognition and treatment can significantly alter the disease course. Furthermore, the use of standardized assessment tools, such as the GBS disability scale, will further aid in monitoring disease progression and guiding therapeutic decisions. Greater awareness of atypical presentations will enhance early recognition, reduce diagnostic delays, and ultimately improve patient prognosis. This case report also highlights the need for continued research into the underlying mechanisms, particularly in the idiopathic presentation of GBS, to improve diagnostic accuracy and broaden our understanding of its pathophysiology.

## References

[1] Hughes RA, Cornblath DR (2005). Guillain-Barré syndrome. Lancet.

[2] Fokke C, van den Berg B, Drenthen J, Walgaard C, van Doorn PA, Jacobs BC (2014). Diagnosis of Guillain-Barré syndrome and validation of Brighton criteria. Brain.

[3] Estridge R, Iskander M (2015). Understanding Guillain-Barré syndrome. JAAPA.

[4] Jacobs BC, van Doorn PA, Schmitz PI (1996). Campylobacter jejuni infections and anti-GM1 antibodies in Guillain-Barré syndrome. Ann Neurol.

[5] Mori M, Kuwabara S, Miyake M (2000). Haemophilus influenzae infection and Guillain-Barré syndrome. Brain.

[6] Willison HJ, Goodyear CS (2013). Glycolipid antigens and autoantibodies in autoimmune neuropathies. Trends Immunol.

[7] Schonberger LB, Bregman DJ, Sullivan-Bolyai JZ (1979). Guillain-Barre syndrome following vaccination in the National Influenza Immunization Program, United States, 1976--1977. Am J Epidemiol.

[8] Sejvar JJ, Baughman AL, Wise M, Morgan OW (2011). Population incidence of Guillain-Barré syndrome: a systematic review and meta-analysis. Neuroepidemiology.

[9] Webb AJ, Brain SA, Wood R, Rinaldi S, Turner MR (2015). Seasonal variation in Guillain-Barré syndrome: a systematic review, meta-analysis and Oxfordshire cohort study. J Neurol Neurosurg Psychiatry.

[10] Cao-Lormeau VM, Blake A, Mons S (2016). Guillain-Barré syndrome outbreak associated with Zika virus infection in French Polynesia: a case-control study. Lancet.

[11] Wakerley BR, Yuki N (2015). Mimics and chameleons in Guillain-Barré and Miller Fisher syndromes. Pract Neurol.

[12] Umapathi T, Tan EY, Kokubun N, Verma K, Yuki N (2012). Non-demyelinating, reversible conduction failure in Fisher syndrome and related disorders. J Neurol Neurosurg Psychiatry.

[13] Uncini A (2012). A common mechanism and a new categorization for anti-ganglioside antibody-mediated neuropathies. Exp Neurol.

[14] van den Berg B, Walgaard C, Drenthen J, Fokke C, Jacobs BC, van Doorn PA (2014). Guillain-Barré syndrome: pathogenesis, diagnosis, treatment and prognosis. Nat Rev Neurol.

[15] Asahina M, Kuwabara S, Suzuki A, Hattori T (2002). Autonomic function in demyelinating and axonal subtypes of Guillain-Barré syndrome. Acta Neurol Scand.

[16] Hughes RA, Swan AV, van Doorn PA (2014). Intravenous immunoglobulin for Guillain-Barré syndrome. Cochrane Database Syst Rev.

[17] Hughes RA, Swan AV, van Koningsveld R, van Doorn PA (2006). Corticosteroids for Guillain-Barré syndrome. Cochrane Database Syst Rev.

[18] Huizinga R, van den Berg B, van Rijs W (2015). Innate immunity to Campylobacter jejuni in Guillain-Barré syndrome. Ann Neurol.

